# Ring 18 chromosome associated with cleft palate: case report and comprehensive literature review of clinical symptoms

**DOI:** 10.1186/s13023-024-03505-2

**Published:** 2024-12-20

**Authors:** Dominika Matyskova, Michaela Richtrova, Alzbeta Novotna, Olga Koskova

**Affiliations:** 1 Department of Burns and Plastic Surgery, University Hospital Brno, Jihlavska 20, Brno, 62500 Czech Republic; 2 Faculty of Medicine, Masaryk University, Brno, Czech Republic; 3 Department of Pediatric Anesthesiology and Intensive Care, University Hospital Brno, Brno, Czech Republic

**Keywords:** Ring 18 chromosome, Cleft lip and palate, Acellular dermal matrix, Anesthesia in children

## Abstract

**Background:**

Ring 18 chromosome is a rare chromosomal aberration associated with a wide range of symptoms affecting all organ systems. One possible symptom associated with this condition is an orofacial cleft. However, to date, there are very few reported cases where the cleft has been surgically treated.

**Case Description:**

In our case study, we present a female patient with Ring 18 chromosome who underwent cleft palate surgery at 14 months of age. Subsequently, a reoperation of the palate was necessary due to wound dehiscence. For the secondary reconstruction of the palate, the acellular dermal matrix (ADM) MatriDerm® was used to improve healing. The cleft palate surgery progressively improved her ability to take in food, allowing a transition from nasogastric tube feeding to oral intake.

**Results:**

This is only the fourth reported case of a child with Ring 18 chromosome undergoing surgical correction of an orofacial cleft. Additionally, it is one of the first cases where an ADM MatriDerm® was used in the surgical correction of a cleft palate. In this study, we also present a comprehensive literature review, providing an overview of the various symptoms associated with this syndrome.

**Conclusion:**

Cleft palate surgery had a very positive effect on improving food intake in the patient with Ring 18 chromosome. The use of an acellular dermal matrix during the secondary cleft palate surgery led to improved healing and a good outcome.

## Introduction

Ring chromosomes refer to an uncommon structural abnormality affecting approximately 1 in 30–60,000 newborn children [1]. They are present among all human chromosomes, although their occurrence is uneven [2]. Typically, ring formations occur as de novo mutations, although familial transmission has also been reported, accounting for an estimated 5.6% of all cases, with approximately 1% of cases occurring in the general population [3]. The ring 18 chromosome mutation is relatively common among all ring chromosomes, with literature suggesting an occurrence rate of as high as 10–12% for ring 18 and 20 chromosome, making them among the most prevalent types [2]. Cleft palate is a common symptom associated with chromosomal abnormalities involving chromosome 18 [4]. However, to the best of our knowledge, only seven reported cases of cleft palate (CP) in patients with Ring 18 chromosome have been documented [5–11].

The aim of our work is to describe the treatment management of a patient with Ring 18 chromosome, who was diagnosed with a cleft palate and underwent cleft palate procedures. Surgical intervention is the only effective treatment for a cleft palate. However, in patients with Ring 18 chromosome syndrome, this procedure can often approach the limits of indication criteria for reconstructive surgery due to the severity and variability of other associated symptoms. The objective of this study was to identify published cases of patients with Ring 18 chromosome syndrome who also presented with cleft palate and to document the timing of their surgical treatment. To support the decision-making process for cleft palate surgery in these patients, we provide a comprehensive and detailed synopsis of the phenotypic spectrum associated with Ring 18 chromosome reported in the literature to date.

## Case report

We present the case of a 4-year-old girl with Ring 18 chromosome, including cleft palate, Pierre-Robin sequence, microcephaly, epilepsy, coagulopathy, and hearing disorders (the patient’s parents provided a Consent to Publish declaration). She was born in the 41st week of gestation, weighing 2960 g (> 3rd percentile), and measuring 44 cm in length. This was her mother’s first pregnancy, with no prenatal pathology detected, and the mother’s age at delivery was 34 years. Due to labor not progressing, a cesarean section was performed.

Following birth, the patient experienced oligohydramnios and presented in a shocked, atonic, and asphyxiated state, with Apgar scores of 2-4-6, desaturation with the necessity of administering oxygen therapy. After one and the half months, a tracheostomy was performed due to respiratory insufficiency. During the postnatal hospitalization period, she was diagnosed with purulent meningitis and received a combination of antibiotic therapy. Subsequently, seizures were observed in the neonatal period, and due to persistent seizures antiepileptic therapy was initiated. During the first year of life, the patient suffered from thrombosis of the cranial veins and anticoagulation therapy was administered for 3 months.

In the first few months of life, she was orally fed, but then tracheostomy installation led to feeding difficulties. Consequently, she was fed via nasogastric tube for an extended period, as the parents declined percutaneous endoscopic gastrostomy (PEG) installation.

### Cleft palate surgery

**Fig. 1:**
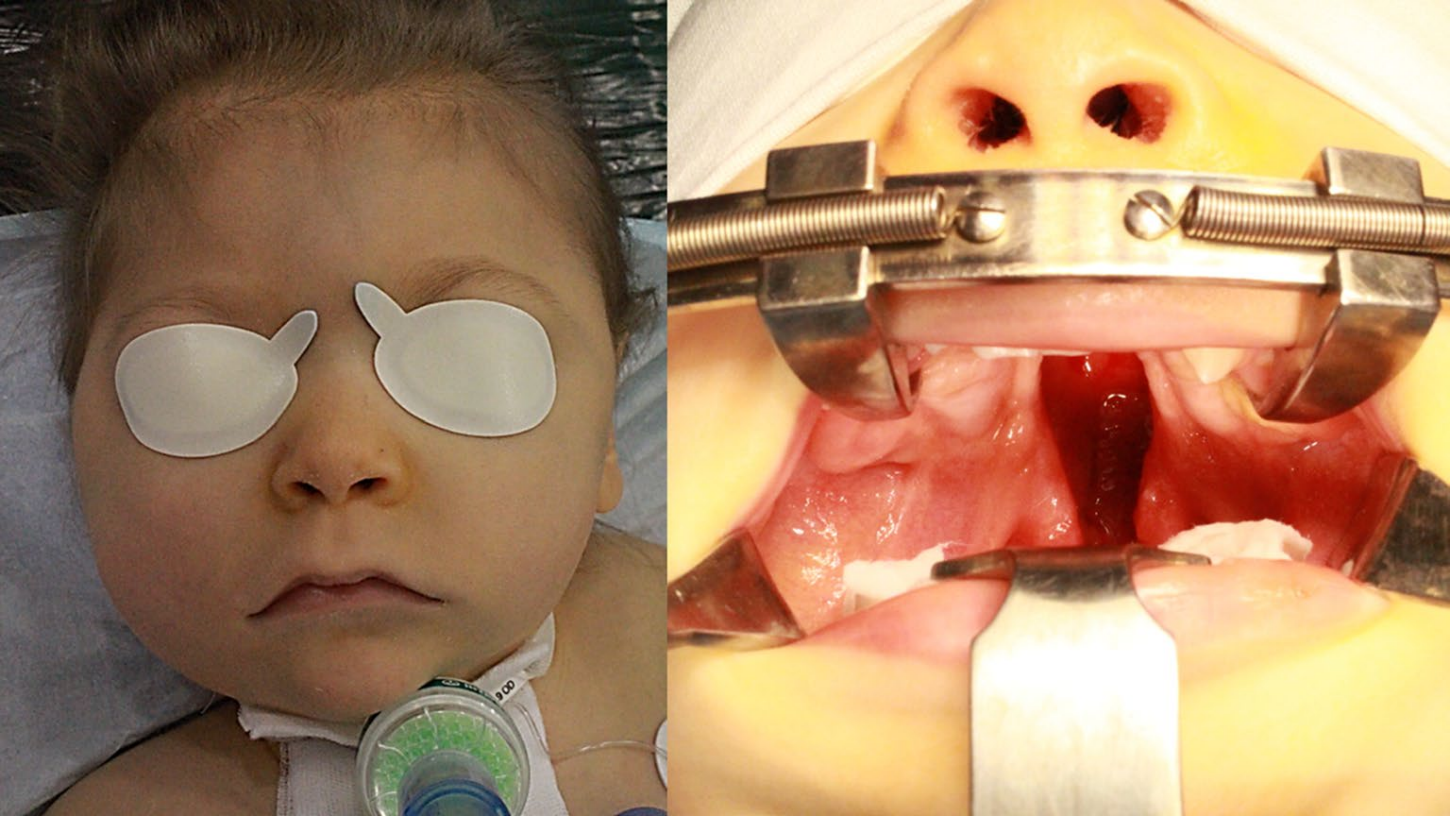
Patient with Ring 18 chromosome and cleft palate

At the age of 14 months, the primary surgery for the cleft palate was scheduled (Fig. 1). Prior to the procedure, microbiological examination of oral cavity smears was conducted, and targeted prophylactic antibiotic therapy with amoxicillin-clavulanate was applied. We performed primary palatoplasty (the von Langenbeck technique) and micro-otoscopy with insertion of ventilation tubes. The surgery proceeded without complications and postoperative care was uneventful. However, on the 13th day after the surgery, a respiratory infection developed, resulting in an early wound healing complication and the formation of a 2 cm oronasal fistula on the palate.

Secondary correction was indicated to improve food oral intake and was subsequently performed at the request of the family. Secondary surgery was performed 18 months after the primary procedure when the patient was 33 months old. During preoperative preparation, a lower level of activated partial thromboplastin time (aPTT) was noted as the level of aPTT-ratio was 1.51, and based on hematological recommendations, etamsylate was administered pre- and perioperatively. As with the primary surgery, microbiological screening of oral and nasal cavity was performed and due to the extensive microbial presence, targeted prophylactic antibiotic therapy with piperacillin-tazobactam was necessary. Closure of the oronasal fistula at the junction of the hard and soft palate was achieved by elevating mucoperiosteal flaps and suturing them in the middle of the defect. Due to tissue fragility, an acellular dermal matrix (ADM) of bovine origin, MatriDerm®, was implanted as a barrier between the nasal and oral mucosa, fixed to the nasal mucosa with absorbable sutures. The healing process following the secondary surgery was favorable without any complications. Successful closure of the palate defect led to improved food intake for the patient, allowing for the removal of the nasogastric tube and transition to full oral intake. In consideration of the patient’s severe syndromic condition, the primary goal was not to improve speech. However, enabling oral feeding was beneficial for both the family and the patient.

### Literature review

**Fig. 2:**
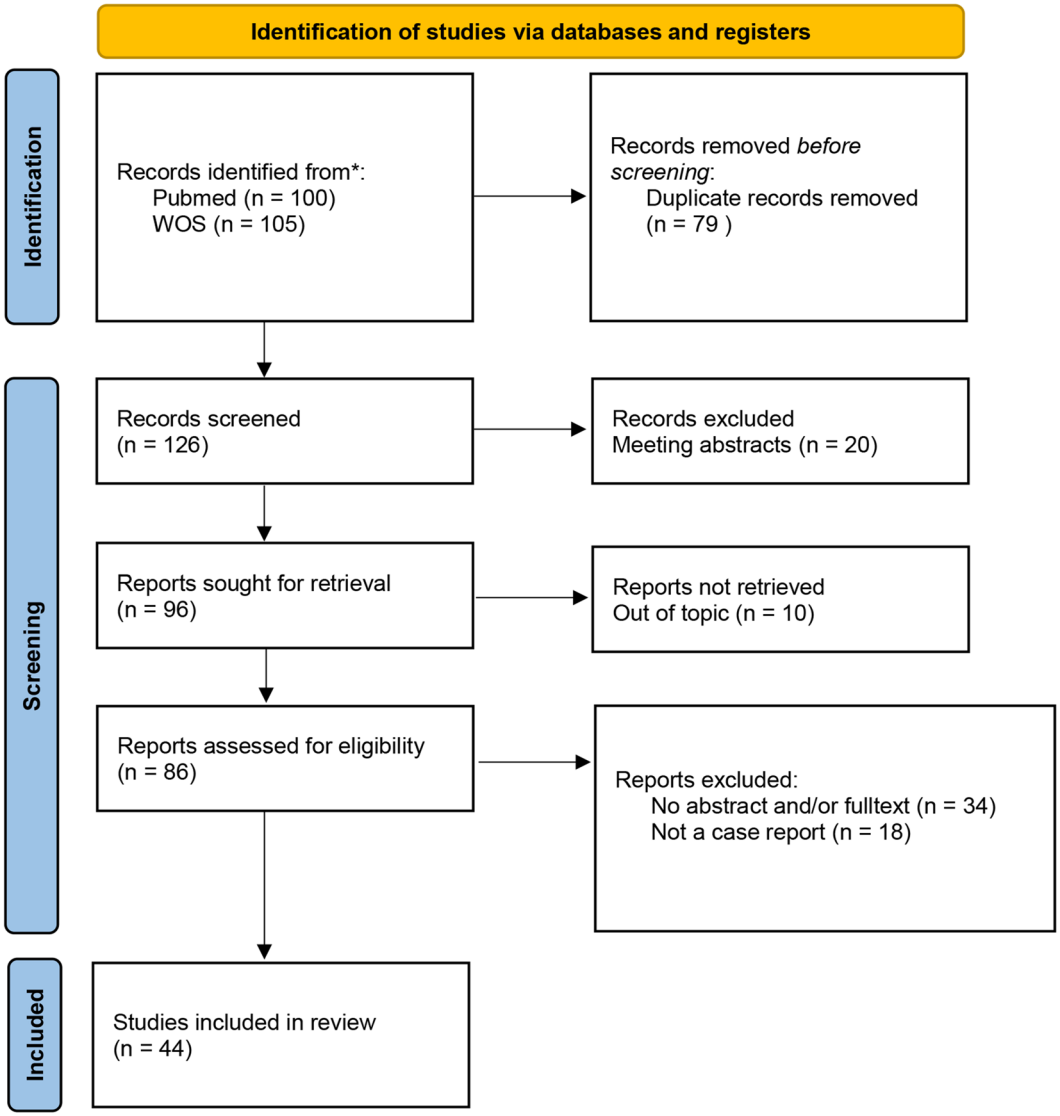
PRISMA flowchart of systematic literature review of Ring 18 chromosome symptoms

In this study, we provided an overview of symptoms previously described in relation to Ring Chromosome 18 Syndrome. A review was conducted, which included all case reports documenting symptoms associated with this syndrome. Publications were searched in the PubMed and Web of Science (WOS) databases, with the search query using the keywords “Ring,” “Chromosome,” and “18” in the title of the article. The search query was formulated as follows: ((ring[Title]) AND (chromosome[Title])) AND (18[Title]). A total of 126 articles were identified using this search strategy. Exclusion criteria included meeting abstracts, articles unrelated to Ring Chromosome 18 Syndrome, review articles describing more than one patient, articles lacking a case report, and articles without available abstracts and/or full texts. Ultimately, 44 articles were included in the review. The detailed selection process is illustrated in the PRISMA flowchart (see Fig. 2). Symptoms reported in the articles for patients with Ring Chromosome 18 Syndrome were extracted and summarized in Table 1.

**Table 1:** Symptoms associated with Ring 18 chromosome

Organ systems	Symptoms
Craniofacial	microcephaly and plagiocephaly [9, 11, 18, 23, 26, 27, 29, 30, 34, 35, 40–44], hypertelorism [5, 6, 9, 22, 29, 34, 41, 43, 45–48], low-set ears or other anomaly of ears [7, 22, 27, 34, 41, 43, 44, 48–53], epicantal folds [5, 7, 9, 26, 27, 30, 34, 45–47, 53, 54], blepharoptosis [22, 48, 52, 54], ankyloglossia or macroglossia [30, 42, 55], other facial abnormalities including clefts [9, 11, 18, 25, 27, 29, 40, 42, 43, 45–51, 53, 54, 56, 57]
Musculoskeletal	fingers malformations [6, 7, 9, 22, 28, 35, 36, 40, 42, 43, 45, 47, 49, 51, 58], scoliosis [42, 54], bilateral subluxation or dysplasia of the hips [22, 54], pectus excavatum [48], other skeletal abnormalities (e.g. spinal anomaly, radial dysplasia, pes equinovarus, pes planus or club feet, short neck, polydactyly etc.) [5, 9, 11, 22, 24, 27, 30, 35, 36, 40–42, 45, 47, 48, 50, 53, 55, 58]
Cardiovascular	enlarged heart [11, 47], heart murmur [7, 11, 29, 30, 58], septal defect [5, 9, 29, 34, 46, 47], patent ductus arteriosus [46–48], dilatation of ascending aorta [46], pulmonary hypertension [46], congenital cyanotic heart disease with aortic-pulmonary collaterals [54], subaortic stenosis [43], valve insufficiency [47], complex heart defect [50], other cardiac malformations [5, 9, 59]
Hormonal	Growth hormone deficiency [7, 37, 38, 52, 54, 60], short stature [22, 26, 27, 29, 30, 34, 37, 40, 45, 48, 49, 53–55, 60] and delayed growth [6, 18, 23, 35, 42, 59], primary hypoparathyroidism [39], primary hypothyroidism [7, 11, 26, 27, 29, 33, 40, 45, 46, 52, 54, 59], insulin dependent diabetes [26, 29], hypogonadotropic hypogonadism [37], absence of breast development [49], delayed puberty [52], premature ovarian failure [52]
Autoimmune diseases	Crohn’s disease and SLE [23], Rheumatoid arthritis [11, 23, 54]
Eye	nystagmus [35, 55], microphthalmia [27], strabismus [7, 22, 27, 34, 46, 48, 55], hypermetropia [46], astigmatism [46], esotropia [46], cataracts [52]
Ear	ear canal atresia [5, 30], external auditory tubes stenosis with hear loss [5, 30, 46, 47, 52, 54, 58]
Respiratory	interstitial lung disease, pulmonary fibrosis, bronchiectasis [11, 52], sleep apnea [55], severe pulmonary stenosis [53], dyspnea [46], laryngomalacia with stridor [46], congenital left lobar emphysema [7]
Urogenital	ambiguous genitalia [35], cryptorchidism [24, 27, 36], hypospadias and urethra anomalies [29, 36], micropenis [9, 34, 37], vesicourethral reflux [36, 46], hypoplasia of clitoris and anterior fusion of labia minor [30]
Skin	vitiligo [11, 26], alopecia and ophiasis [51, 53], sparse hair and dry skin [30, 45, 46], Blaschkoid hypermelanosis [44], atopic dermatitis [59]
Mental and development	intellectual disability and developmental delay [6, 7, 18, 22–30, 34, 40, 42–45, 47, 48, 52, 55, 58, 61]
Neurological	hyperactivity [18, 25], stereotypic movements [18, 27], hypotonia [7, 9, 23, 26, 30, 34, 42, 44, 45, 47, 49, 55], epilepsy and seizures [18, 25, 41], autism [31, 52], abnormal myelination [26, 34, 42, 54, 55, 62], extensor hypertonus of lower extremities [22]
Immunological	agammaglobulinemia [22] and other disorders of immunoglobulin function [11, 26, 32–34, 52, 54, 58, 59]
Gastrointestinal	anal atresia [41], celiac disease [41], gastroesophageal reflux [7, 42], lactose intolerance [26]

## Discussion

Our paper represents only the fourth published case of a surgical reconstruction of a cleft palate in a patient with Ring 18 chromosome. Among the few documented cases of this nature, this report stands out as the first to provide a comprehensive overview of the surgical approach used to treat cleft palate in patients with this genetic condition.

### Genetic aspects of ring chromosomes

It is assumed that ring chromosomes arise either from a direct fusion of telomeres or from a breakage of the distal parts of chromosomal arms, which can occur during cell division. This is often followed by intrachromosomal fusion, resulting in the formation of a circular structure. During the process, loss of genetic material commonly occurs, which can lead to deletion syndromes with phenotypic variations depending on the extent of deleted regions [1, 12, 13]. Ring chromosomes can be present as germline alterations that are either inherited or arise de novo. Alternatively, they can form through somatic alteration, resulting in mosaicism [14, 15]. Upon suspicion based on clinical presentation, the diagnosis of ring chromosomes typically involves methods such as karyotyping, fluorescence in situ hybridization (FISH) or other cytogenetic or molecular-cytogenetic techniques, which are often performed using peripheral blood lymphocytes [16].

### RING 18 chromosome

Ring 18 chromosome or R(18) was first described by Wang et al. in 1962, being among the first ring chromosomes reported in humans [17]. Since then, more than 70 cases have been reported [18]. In 1963, de Grouchy was one of the first researchers to define the ring 18 phenotype as a combination of features seen in both 18q and 18p deletion syndromes, with a high variability in phenotypic expression and multisystemic involvement [19]. Given that the Ring 18 chromosome can be associated with deletions on both the 18q and, less frequently, the 18p arm, patients present with features of the respective deletion syndromes. Some patients exhibit a combination of both deletion syndromes, often resulting in a more severe phenotype than isolated deletions of 18q or 18p alone [20]. Interestingly, most patients with Ring 18 chromosome are found among females, usually presenting with less severe phenotype than isolated 18q deletion syndromes [20, 21].

Ring 18 chromosome has so far been associated with many associated symptoms, typically it is manifest with short stature, mental retardation, microcephaly, neurological disabilities, and various amount of dysmorphic features, such as hypertelorism, micrognathia, and epicanthic folds [22].

A wide range of symptoms affecting multiple organ systems associated with Ring chromosome 18 syndrome or Ring 18 mosaicism are described in the literature (see in Table 1). Among the most frequently mentioned are intellectual disability and developmental delay [6, 7, 18, 22–42];. neurological abnormalities [7, 9, 18, 22, 23, 25–27, 30, 31, 33, 35–37, 39, 40, 43–47]; and immunological abnormalities [11, 22, 26, 31, 39, 41, 46, 48–50]. Ring chromosome 18 syndrome is also characterized by craniofacial abnormalities [5–7, 9, 11, 18, 22, 23, 25–27, 29–40, 44–46, 51–57], and other musculoskeletal abnormalities [5–7, 9, 11, 22, 24, 27, 28, 30, 32–34, 36–38, 40, 41, 44–46, 51, 53–55, 58]. Autoimmune diseases [11, 23, 46], growth hormone deficiency [7, 39, 46, 59–61], and other hormonal imbalances have been observed in association with Ring 18 chromosome [7, 11, 26, 27, 29, 49, 59, 36–52, 45, 39, 46, 50].

### RING 18 and cleft lip/palate

It is widely accepted that cleft palate (CP) is one of the possible symptoms of 18q- deletion syndrome. However, to the best of our knowledge, there have only been 7 reported cases of a CP in a patient with Ring 18 chromosome (see in Table 2) [5–11]. Although isolated cleft lip (UCL) [24], cleft lip and alveolus (UCLA) [57], or an velopharyngeal insufficiency [63] have been reported as well.

**Table 2:** Cleft palate and Ring 18 chromosome

Publication	Date of publication	Type of cleft	Surgical procedure	Age during surgical procedure
Kalker et al. [5]	1988	CP	Data are not available	Data are not available
Tavin et al. [6]	1994	Submucous CP	No	X
Dobos et al. [10]	2004	UCLAP	Yes	Data are not available
Thomas et al. [7]	2006	CP	No	X
Souraty et al. [8]	2007	UCLAP	Yes, cleft lip and palate (one procedure)	2.5 years
Chen et al. [9]	2010	Cleft palate	Data are not available	Data are not available
Chau et al. [11]	2017	Cleft palate	Yes	3 years

The first described case of a cleft palate in patient with ring 18 chromosome was reported in 1988 in Frankfurt, Germany [5]. The 7-months-old female also showed features of Van der Woude syndrome, with fistula of the lower lip and other facial abnormalities.

In subsequent years, cases were reported across different regions across the world, including USA [6, 11], Hungary [10], Brazil [7], Lebanon [8] and Taiwan [9]. However, among all reported patients with CP, surgical reconstruction was described in only three of them [8, 10, 11].

First patient was a 14-months-old girl with a unilateral cleft lip and palate (UCLP), with the reconstruction performed after the age of 14 months [10]. The second case was a girl in Lebanon in 2007 with the same condition, who also presented with growth retardation, developmental delay, neurological abnormalities, and various abnormal facial features. The cleft palate surgery was performed at the age of 6 months of the patient [8]. The third case involved a 32-year-old female refugee from the Dominican Republic, who was treated in the USA for pneumonia. During the assessment of her medical history, it was noted that she underwent cleft palate surgery at the age of 3 years in the Dominican Republic. However, residual posterior cleft palate and split uvula were present [11].

### Use of acellular dermal matrix in palatoplasty

Tissue quality is generally worse in syndromic cleft palate patients [7]. ADM is generally used to improve wound healing after various surgeries in oral cavity including cleft palate surgery [64–69]. In cleft palate surgery, the use of ADM was described both in primary palatoplasty and in secondary operations [64], specifically authors have observed the benefit of use of ADM to cover a defect in the nasal mucosa [66] or to solve oronasal communications [65, 67, 70].

Numerous types of ADM have been developed by manufacturers worldwide and different types of ADM have been used in different areas during surgery in oral cavity [64]. So far, many types of ADM have been used in reconstructive surgery in oral cavity, specifically human ADM Alloderm®, DermaMatrix® and Porous®, porcine ADM Protexa® and bovine ADM Heal-All® and MatriDerm®. MatriDerm® belongs to acellular dermal matrix (ADM) of bovine origin and it was previously successfully used to reconstruct defect of the palate in patient with cleft palate in Spain [65]. In our case, ADM MatriDerm® was used to improve healing after secondary cleft palate surgery performed to repair oronasal fistula.

## Conclusion

Ring 18 chromosome is a very rare genetic disease associated with many congenital developmental anomalies. So far, 7 cases of patients with this disease associated with orofacial cleft have been described in the literature. We present the case of a patient with UCLP associated with the Ring 18 chromosome, who underwent primary cleft palate surgery at 14 months and secondary surgery at 3 years of age while ADM MatriDerm® was used to improve wound healing. The surgical treatment of a cleft palate in patients with Ring 18 chromosome is indicated based on an individual assessment of the patient’s overall condition.

## Data Availability

The datasets used and/or analyzed during the current study are available from the corresponding author on reasonable request.
